# BEST INTENTIONS OR BEST PRACTICE? HOW DO STAFF PERCEIVE BEST CARE FOR PEOPLE WITH DEMENTIA IN TAIWAN?

**DOI:** 10.1093/geroni/igz038.1870

**Published:** 2019-11-08

**Authors:** Chih-ling Liou

**Affiliations:** Kent State University, North Canton, Ohio, United States

## Abstract

Care of people with dementia is a global issue, and the quality of dementia care in the long-term care facility is of concern internationally. The Taiwanese government seeks to expand the availability of adult day services (ADS) to meet the needs of an aging population with dementia; however, research about care in ADS in Taiwan is limited. This study aims to investigate how ADS staff perceptions of best care affected care delivery and clients’ quality of life. A focused ethnographic method was employed to collect data through 480 hours of participant observation and 31 staff interviews at three centers. Content analysis was used to analyze the data. The findings revealed a contradiction on how staff perceived best care and what they really performed at center. Thirteen staff members who were interviewed identified that in their view the best care is to “put yourself in the shoes of the client,” and another nine staff members chose “keep clients happy” as the best care. From the observation data, however, the staff performed the “good care” by forcing the clients to follow their orders absolutely unless the clients refused violently. Staff-led activities were structured as tasks to be completed rather than activities to be enjoyed. The clients complained about the bureaucratic management that shaped their lives at centers but could only endure or accept. To support clients’ quality of life and improve the care quality, ADS staff need to be educated more with the evidence-based best care for people with dementia.

